# BK Virus-associated Nephropathy in a Patient with T Cell Leukemia/Lymphoma Syndrome

**DOI:** 10.7759/cureus.5070

**Published:** 2019-07-02

**Authors:** Baoqiong Liu, Lingbin Meng, Xuan Guan, Yifeng Yang, Manoucher Manoucheri

**Affiliations:** 1 Internal Medicine, AdventHealth, Orlando, USA; 2 Internal Medicine, St. Vincent’s Medical Center, Bridgeport, USA

**Keywords:** collapsing glomerulopathy, bk virus, t cell leukemia/lymphoma syndrome

## Abstract

BK virus-associated nephropathy develops in renal transplant patients with the main manifestation of tubulointerstitial nephritis or ureteral stenosis. Nephrotic syndrome is a rare manifestation of BK virus-associated nephropathy. Here we report a case of a 69-year-old female presenting with nephrotic and nephritic syndrome related to BK virus infection. Kidney biopsy revealed severe acute tubular injury, collapsing glomerulopathy, and focal microangiopathic changes. Four weeks of leflunomide and intravenous immunoglobulin (IVIG) resulted in the recovery of kidney function and improvement of proteinuria.

## Introduction

BK virus is a member of the polyomavirus family. Primary BK virus infections occur early in childhood via oral or respiratory exposure [1]. Most primary infections are asymptomatic, or with mild symptoms. The virus can disseminate to kidneys and persists for lifelong [2, 3]. It is reported that up to 60-80% of the population contains a latent form of this virus [4].

BK virus can replicate even when the body undergoes immunosuppression. BK virus replication is associated with hemorrhagic cystitis in bone marrow transplant recipients, ureteral stenosis and interstitial nephritis in patients with renal transplant [5, 6].

We herein report a rare case of histologically-confirmed collapsing glomerulopathy associated with BK virus infection in the native kidneys of a patient with acute T cell leukemia/lymphoma syndrome. 

## Case presentation

A 69-year-old African-American female presented with one month of worsening bilateral lower extremity swelling/edema. She was non-oliguric upon arrival. Her past medical history was significant for adult T-cell leukemia/lymphoma, which was diagnosed two months prior to admission. She was initially treated with interferon alfa-2b and lamivudine, which were discontinued due to prolonged neutropenia. Then, she underwent two cycles of cyclophosphamide, doxorubicin, vincristine, and prednisone (CHOP) regimen chemotherapy, which was complicated by an episode of acute kidney injury that quickly resolved. She has a family history of hypertension. 

On admission, her blood pressure was 135/78 mmHg. Physical examination was otherwise largely unremarkable except for bilateral lower extremities pitting edema up to the knees.

Initial workup showed significantly elevated serum blood urea nitrogen (BUN) and creatinine at 78 mg/dL and 3.9 mg/dL respectively, and low serum albumin level of 1.7 g/dL. Hemoglobin was 8.2 g/dL and mean corpuscular volume (MCV) was 90.5 fL. Her urinalysis showed albumin was 3+, blood was 2+, red blood cells (RBCs) were 8/HP, white blood cells (WBCs) were 42/HP. She was found to have severe nephrotic range proteinuria with a urine protein/creatinine ratio of 10.7. High urine and serum BK viral load were detected. Autoimmune workup including anti-double-stranded DNA (anti dsDNA), antinuclear antibody (ANA), antineutrophil cytoplasmic antibodies (ANCA) and complements levels were within the normal range. Hepatitis panel and HIV 1/2 antibodies were negative. Thyroid-stimulating hormone (TSH) and parathyroid hormone (PTH) were intact. Ultrasound revealed increased echogenicity of both kidneys. Echocardiogram manifested acute declination of left ventricular systolic function with an ejection fraction of 25-30%, which was well-preserved for two months.

**Figure 1:**
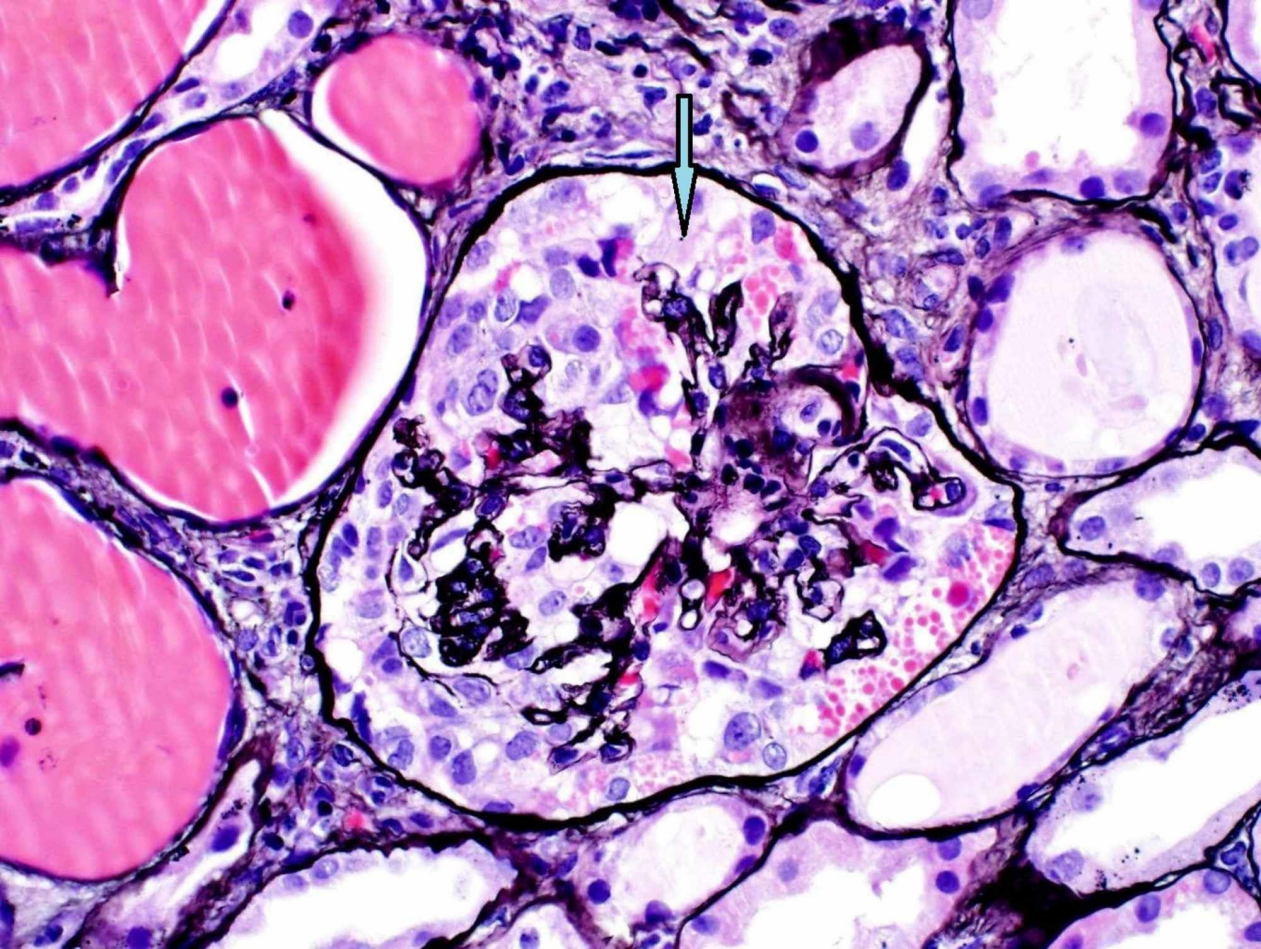
Renal biopsy shows collapsing glomerulopathy.

**Figure 2:**
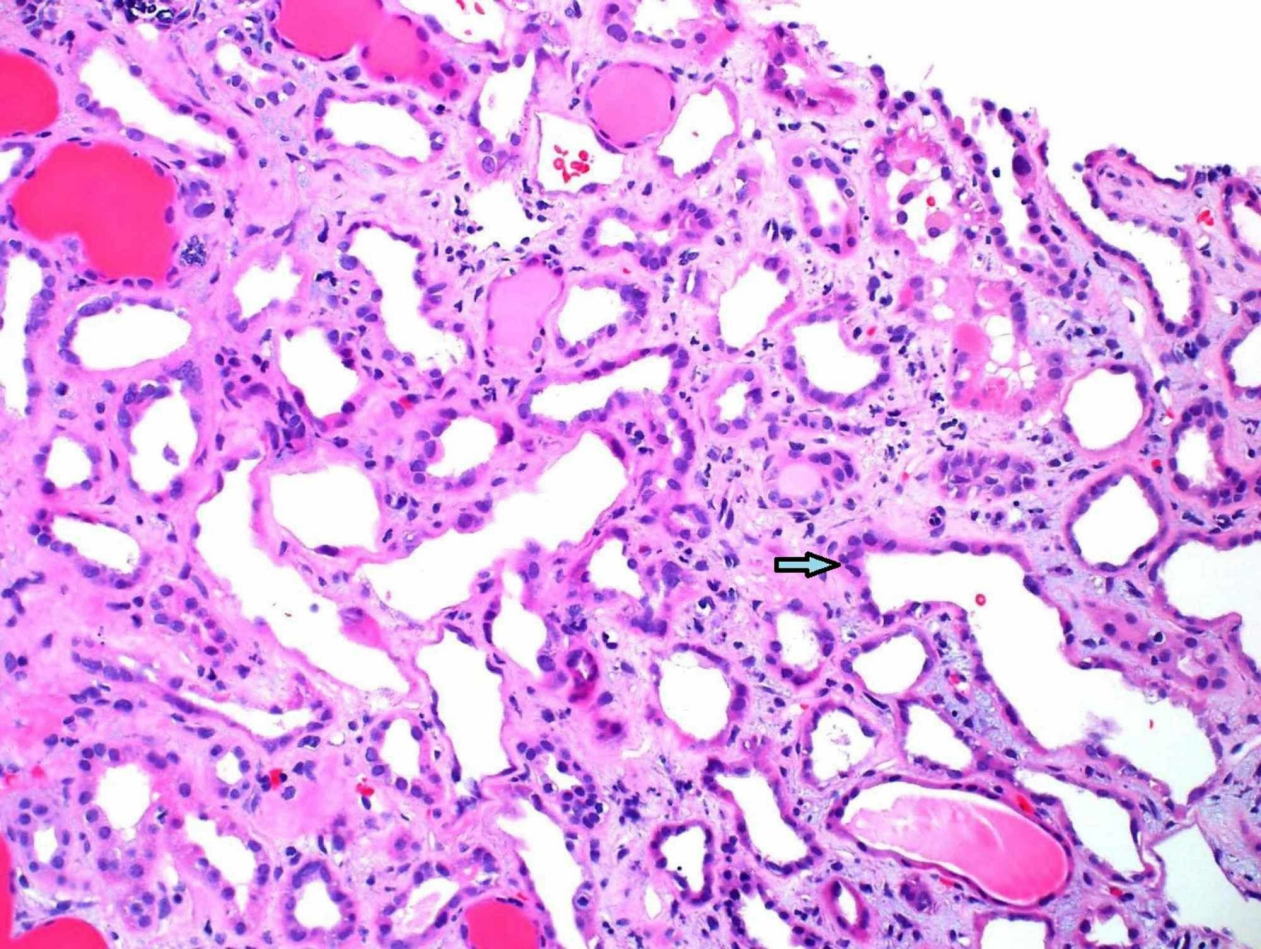
Renal biopsy shows tubular injury and lymphocytic infiltration.

For further investigation, a kidney biopsy was performed, demonstrating collapsing glomerulopathy (Figure 1) and moderate to severe tubular necrosis (Figure 2). Lymphocytic invasion and nuclei were noted in tubules, suggestive of viral tubular invasion/nephropathy. No cytomegalovirus (CMV) was observed on repeat review of the renal parenchyma. Congo red stain was negative. 

The patient was diagnosed with nephropathy associated with BK virus infection in the kidneys. Interstitial nephritis related to Bactrim® may also be the cause but no interstitial nephritis was observed in this patient’s biopsy.

The patient received intravenous immunoglobulin (IVIG) 2 g/Kg and Arava® (leflunomide) 100 mg p.o. daily for BK virus-associated nephropathy. A high-protein diet and protein supplements were offered as well. Three weeks of Arava® (leflunomide) and IVIG treatments resulted in resolution of her bilateral lower extremity edema, recovery of kidney function and improvement of proteinuria.

## Discussion

BK virus is highly seroprevalent in humans, but it appears to cause problems only among immunocompromised patients. BK virus-associated nephropathy manifests as an asymptomatic gradual rise in creatinine, and an abnormal urinalysis revealing renal tubular cells and inflammatory cells with tubulointerstitial nephritis [7]. Collapsing glomerulopathy is a morphologic variant of focal segmental glomerulosclerosis, which accounts for about 40% of the nephrotic syndrome in adults [8]. BK virus associated-nephrotic syndrome or collapsing nephropathy is rare, with only two cases reported previously. A 12-year-old boy with end-stage renal disease due to nephronophthisis developed BK virus-associated nephritic-nephrotic syndrome 18 months after a kidney transplant [9]. The other case occurred in a native kidney of a previously healthy 4-year-old boy [10].

In our case, the patient presented as nephrotic/nephritic syndrome, with edema, azotemia, hypoalbuminemia, and heavy proteinuria. Kidney biopsy confirmed collapsing glomerulopathy and tubular injury as the cause. Tubular necrosis with lymphocytic invasion and nuclei were noted in tubules, which was suggestive of viral tubular invasion/nephropathy. Her urine and serum BK viral load were both found to be high. Two to three weeks after anti-BK virus therapy with IVIG and leflunomide, her kidney function recovered to her baseline and her proteinuria significantly decreased to 2 + on urinalysis. According to the time course and clinical scenario, a final diagnosis of BK virus associated-collapsing glomerulopathy and tubular necrosis was made. Although the patient had been taking Bactrim® for prophylaxis, no interstitial nephritis was observed in this patient’s biopsy. However, there might be a component of congestive nephropathy/cardiorenal syndrome in the backdrop of the acute decline of left ventricular systolic function, causing a lack of forwarding blood flow. And her primary hematological disease and race could also put her at risk for collapsing glomerulonephropathy. 

Minimization of immunosuppression upon detection of BK viremia may prevent clinically evident BK virus nephropathy. While there has been no FDA approved treatment for BK virus-associated nephropathy. Generally, it is treated with immunomodulant agent leflunomide, or IVIG together with the reduction of immunosuppression [11]. For BK virus associated-nephrotic syndrome/collapsing glomerulopathy, the immunomodulant agent seems to be helpful in our case. Nevertheless, immunosuppressive treatment resulted in graft failure, renal failure or even death in the two previous cases [9, 10]. Further research is needed to determine effective treatment.

## Conclusions

BK virus load should be examined on patients with nephrotic and or neprhitic syndrome, especially on these undergoing immunosuppression. Reduction of immunosuppression should be considered and carefully monitored if the nephrotic syndrome is associated with BK virus. Further studies are required to determine the effective treatment for BK virus-associated nephrotic syndrome.
